# A Perceptual Structure of Soundscapes in Urban Public Spaces Using Semantic Coding Based on the Grounded Theory

**DOI:** 10.3390/ijerph20042932

**Published:** 2023-02-08

**Authors:** Jingwen Cao, Jian Kang

**Affiliations:** 1 School of Architecture and Urban Planning, Nanjing University, 22# Hankou Road, Gulou District, Nanjing 210093, China; 2 UCL Institute for Environmental Design and Engineering, The Bartlett, University College London (UCL), Central House, 14 Upper Woburn Place, London WC1H 0NN, UK

**Keywords:** soundscape structure, grounded theory, semantic coding, perceived sounds, urban public spaces

## Abstract

The definition of ‘soundscape’ emphasises the perceptual construct of sounds; thus, the mechanism of sound perceptions becomes vital for soundscape evaluations. Using a qualitative approach, this study explored the aspects and processes of sound perceptions and built a perceptual soundscape structure from the insight of sociology. The interview was conducted between January and March 2018, in four urban public spaces. Data reached saturation after 23 participants were interviewed based on the grounded theory approach. Four perceptual aspects of sounds were identified from the semantic coding analysis: sound classification, sound features, psychological reactions, and soundscape preferences. These aspects form a three-level process of perceiving soundscapes: sound classifications, sound appraisals (including sound features and psychological reactions), and finally, judgment (soundscape preferences). Overall, four aspects categorised into three levels of perception make up the soundscape structure. Soundscape preferences are at the most profound level of perception and are informed by the previous three aspects. Soundscape preferences are expressed through descriptive words and narrative ‘image’. The ‘image’ reflects people participating in different activities according to their social background. Social relationships influence soundscape preferences through people’s sound requirements for various activities. The perceptual structure of soundscapes may provide guidance for future soundscape research and soundscape questionnaire design.

## 1. Introduction

The relationship between sounds and humans and the way people perceive the acoustic environment were originally emphasised to stress the ‘degradation’ of the sustainable acoustic environment due to industrial growth and the process of urbanisation [1,2]. Early sustainable acoustic studies focused on various urban noise problems and noise management [3], concerning its negative effect on mental health and well-being [4,5,6] as well as the emotional aspect [7,8]. Beyond noise management, some researchers have focused on how people subjectively perceive sounds by considering sounds a ‘resource’ rather than a ‘waste’ [9], and the concept of ‘soundscape’ emerged [10]. ‘Soundscape’ was defined by the International Organization for Standardization [11] to emphasise the perceptual construct of sounds: ‘[the] acoustic environment as perceived or experienced and/or understood by a person or people, in context’. This definition clarifies that soundscapes exist through human perception of the acoustic environment rather than the physical phenomenon (the acoustic environment) itself [12,13].

It was suggested that people perceive sounds through a systematic process [14]. Present studies usually classify the soundscape perception into several elements with the aim to identify the perceiving process [15]. The method of classifying sound sources is considered as the first step of listening [16,17]. Thus, two strategies for sound classification were summarised as ‘descriptive listening’ and ‘holistic listening’ [18]. The former referred to the identification of sound sources or events; the latter is concerned with perceiving the soundscape as a whole without semantic processing, that is, without isolation of specific events. Others analysed soundscapes through the framework of the environmental perception theory [19], and proposed several decisive factors of sound perceptions as person (community), activity, and place, and the interactions between person and place. Sound perceptions of each person are made up by emotion (feelings), cognition (thoughts), and knowledge (meaning). Further on the perceiving process, five dimensions of sound perceptions were generated by Liu and Kang [20] in the urban context from the past to future: soundscape definition, soundscape memory, soundscape sentiment, soundscape expectation, and soundscape aesthetics. These five dimensions capture people’s understanding and psychological needs regarding the urban soundscape. Davies et al. [21] suggested that how individuals perceive sounds is influenced by a cognition process, which consists of three components: sound sources, sound descriptors, and soundscape descriptors. The sound source is referred to as a physical entity; sound descriptors are descriptions of sounds; soundscape descriptors refer to the totality of what is heard. In short, previous studies categorised the key dimensions of sound perceptions, but how these aspects work and the relationships among them are not focused.

Others focused on how sound signals reach the perceptual level. Schulte-Fortkamp and Fiebig [22] outlined five stages of how people perceive sounds: the acoustics of the sound(scape), the initial perception, a negotiation process internal to the listener, psychological reactions, and behavioural responses. Similarly, the perceptual structure of soundscapes defined in the International Organization for Standardization (ISO) [23] explained how physical sounds are perceived and understood by people from the acoustic environment through auditory sensation and its interpretation to response and outcome. This process enables people to process auditory signals into meaningful information so as to understand the acoustic environment. Then, people would react with responses and outcomes towards the perceived sounds. Above studies, especially the perceptual construct of soundscape summarised by ISO, described a general sound perception process based on psychological theories. They identified the key perceptual stages of soundscape and explained the perceptual process from physical sounds to the perceptual sounds. Among those perceptual stages, the stage of ‘interpretation of auditory sensation’ refers to the processing of the auditory signal to useful information, which results in auditory understanding and awareness. Listeners’ awareness and understanding towards the acoustic environment is shaped by his or her social context, which has not been carefully discussed in this structure. Considering the social function of urban public spaces, it is important to explore from which dimensions listeners perceive sound in urban public spaces and how the process occurs through the insight of sociological research.

Based on the previous studies, this study aimed to explore further the mechanism of how sound signals are interpreted to sound awareness by the urban public space users with a sociological insight. Two research aims were defined as (1) to figure out the dimensions of soundscapes in urban public spaces from the perspectives of space users; (2) to figure out the interrelationships among the soundscape elements so as to form the perceptual process. By investigating these two issues, a perceptual structure of soundscapes with different perceiving stages in urban public spaces can be obtained.

## 2. Methods

### 2.1. Research Design and Process

The grounded theory (GT) approach has been largely used in the soundscape research to generate the understandings of sound perceptions. The GT approach is a research methodology that results in the production of a theory to explain the systematically collected data [24,25]. Researchers are believed to generate a valid theory from subjects’ own language systems consisting of interrelated concepts through this approach [26].

According to the GT approach, to collect abundant research data, in-depth interviews are the key method. In this study, interviewees were selected through the convenience sampling. Convenience sampling is applied when the study site is identified but the sample size is not known. Following this sampling method, the researchers recruit samples at a targeted location by random—researchers may ask people who are present in the street, public buildings, or in a workplace [27]. Compared to the convenience sampling, random sampling is adopted when the sample size is determined, and the researcher selects each sample randomly from the population [28]. In this study, convenience sampling was chosen as the research site is defined as urban public spaces. The researchers recruit samples who are present at the site, randomly in a clockwise order. After the potential interviewee agreed, a short conversation was conducted to inform them of the aims and objectives of the study and to inform them that the interview would take a relatively long time. The normal perceptibility (i.e., normal hearing abilities and sight) required in this study was identified through self-assessment during this conversation. Those willing to be interviewed would be asked to experience the acoustic environment of the site in order to collect their insights into the sound of the site. There were two phases of recruitment, both of which were approved by the Ethics Committee from the University of Sheffield (019886). In the first phase, a pilot study was conducted involving ten participants recruited in two urban public spaces—Guanqian Square and Central Park Square in Suzhou, China—during January 2018. After the research of the pilot study, it was found that site differences added diversities to the sound types as well as the public space user types. Sound perceptions of those respondents were highly correlated with the context of the site and their social background. Thus, two more researcher sites were included: Peace Garden and Barkers’ Pool in Sheffield, UK from January to March, 2018.

During the research, the researcher took notes to record the sound sources heard in the sites and measured the SPL (sound pressure level). Sound sources heard in the sites included the sound of wind, bird, music, cars, and various sounds produced by people, such as talking, steps, children, etc. SPL was measured by a sound level meter (01 dB Solo, Limonest, France). The measurements were conducted during each interview. The range of the A-weighted equivalent sound pressure levels for the four sites were listed as 65.0 dB~68.5 dB (Guanqian Square, Suzhou, China), 70.5 dB~75.8 dB (Central Park Square, Suzhou, China), 68.5 dB~70.8 dB (Peace Garden, Sheffield, UK), 59.1 dB~60.7 dB (Barker’s Pool, Sheffield, UK). Both sound sources and SPL information were found to be representative of the soundscape of urban public spaces based on the previous studies [29]. Data were recorded and initially analysed along with the interview process, and when no new content emerged, interviews ended. This process is defined as ‘data saturation’ which marks the end of the interview [24,30]. Each interview included several unstructured, open-ended questions and lasted for about 30 min, depending on the length of the interviewee’s responses. A total of 23 participants were recruited, as shown in Table 1: 13 male and 10 female. The age of the respondents ranged from 18 to 70 with 6 young participants under 20, 12 adults between 36 and 50, and 5 older participants between 55 and 70.

### 2.2. The Structure of Interview

Table 2 shows the structure of interview questions accompanied by the question aim. Firstly, participants’ background information was obtained, including age, occupation, companion types, and activity type. Gender information was identified during the talk. The second part included the sounds they heard in their daily routine and the sounds they heard in the site and how they described these sounds. This part of the questioning allows the researcher to observe the content and processes by which the participants perceive and understand the sounds. The third part involved subjective understandings, asking participants about their feelings and preferences about the sounds in order to dig deeper into sound perceptions.

### 2.3. Data Analysis

According to the previous practice of the GT approach, the interview data were analysed through semantic coding with the successive use of open, axial, and selective coding [31]. Those three types of coding were manipulated by the process shown in Table 3. Raw data were transferred and analysed through the software of Nvivo 12. Firstly, to deal with the massive raw data, the process of sorting memos and labelling was conducted following the concept of ‘open coding’. Sorting memos refers to the process of collecting interview contents according to the questions and sorting them into key phrases. Labelling enables the sorted text to be identified and merged in short concise sentences. A total of 176 items were labelled at this stage, named as a1, a2, a3, …. Then, they were conceptualised and cut down to 105 items during the second phase of open coding (aa1, aa2, aa3, …). Axial coding occurred simultaneously with the second phase of open coding; this allowed the classification of unstructured data into concepts and categories. After this, 105 items generated 55 categories shown as: A1, A2, A3, …. To identify the main categories, selective coding was adopted reviewing and analysing the interrelationships among those categories. The main categories identified four aspects of sound perceptions shown as AA1, AA2, AA3, and AA4. The relationships among these categories were further considered, according to the categorised data, to form the perception process and build the relationship structure of soundscapes.

## 3. Results

### 3.1. Sound Classifications

With various sounds in the environment, categorising them was the first thing that came to mind when participants were asked to describe soundscape. People tended to recognise sounds within a category, rather than individually. Three kinds of categorising methods emerged: (1) categorising sounds by sound attributes, (2) categorising sounds in the order in which they were noticed, and (3) categorising sounds by the information they conveyed.

#### 3.1.1. By Sound Type

Categorising sounds by type was based on people’s common sense and life experiences. Nature was the most frequently mentioned; this included ‘*sounds of trees, birds, water, and wind …*’ (a29). In contrast to natural sounds, participants categorised the sounds of music and bells as ‘artificial sounds’ (a85). ‘People sounds’ were also mentioned. This included talking, children’s laughing and screaming, and footsteps (a48, a77). The rest were categorised into the group of ‘city sounds’, such as traffic noise, wind between skyscrapers sounds, and store music (a25, a90, a94, a40). Thus, there were four categories of sounds: (1) human sounds: talking, laughing and screaming, and footsteps, (2) natural sounds: trees, birds, water, and wind, (3) instrumental sounds: music and bells, and (4) city sounds: traffic noise, wind from the urban canyon effect, and store music. This classification method represents a basic understanding of sound attributes.

#### 3.1.2. By Attentiveness

As public squares are usually located in the city centre, various kinds of sounds from the city make up a complex acoustic environment. In such a complex environment, the cognitive process would enable people to navigate their surroundings and differentiate between salient and background sounds. The sound of water was mentioned mostly as a foreground sound, and some participants considered it to be so loud as to mask background sounds (a87). Background sound included faraway sounds, such as the sounds from surrounding shops and amusement facilities (a65, a20). It seems that participants generally distinguished between foreground and background sounds based on volume.

Some participants mentioned that background sound had both a negative and a positive influence on the whole sound environment. Background sound could be annoying when it disturbed the overall soundscape; for example, because Guanqian Square is located inside the commercial centre, the annoying ‘*sounds from the amusement park nearby*’ and *‘promotion campaign sounds*’ (a10, a20, a65) were emphasized. Although the background sounds mentioned were quite far away from the square, they were required to fit in the overall soundscape of the square to create a satisfactory sound level. The positive influence included ‘*Water from the surroundings echoes with the water sounds here, which makes a connection*’ (a16). Compared to the negative effect, when the surrounding sounds were positive and in harmony with the foreground sounds, they were considered to have a positive effect.

#### 3.1.3. By Sound Meaning

The various pieces of information contained in sounds were used as a classification method because sound is a medium for conveying information. As information tends to be time-sensitive, information-related sounds were categorised into two types: (1) current information, where listeners could learn about events and situations that were happening at the moment, for example, clock bells providing information about the time (a101) and (2) past information, or sounds associated with memories. For example, typical water sounds in the Peace Garden were reminiscent of people’s memories (a19). Store music was also believed to trigger memories because of some old songs that were played (a20).

In short, classification is fundamental to how people understand sounds. Compared to the classification methods used in previous research, the one used in the present study did not involve delving deep into the physical attributes of sounds, such as strength and fluctuation [29]. The three kinds of categorising methods summarised in this study reflect the fact that people tend to classify sounds merely by content and volume.

### 3.2. Sound Features

Faced with multiple sound sources in the square, participants tended to think of the relationship between individual sounds and the overall sound environment. They concluded that there are two kinds of relationships: the ones between diversity and integrity and between particularity and stereotypes. The first one refers to people’s recognition of the coexistence of multiple sounds and the requirement for those sounds to be harmoniously combined into an integral whole; the other indicates that people had a requirement for the particularity of a sound to identify the square, but they did not want this particularity to exceed their general understanding of the soundscape of the square. Both relationships show how people thought logically and critically about the characteristics of the square’s soundscape.

#### 3.2.1. Diversity and Integrality

Firstly, a wide range of sounds was recognised as a positive feature of the square’s soundscape (a56, a43). In addition to the diversity embodied by multiple sound types, variations in the tone and volume of the same sound can also bring diversity. Fountains were mentioned as showing this kind of diversity, as the changing water flow can bring with it various sounds (a78). Sound tone was mentioned with reference to bird sounds, as some birds can make changeable sounds (a51). People even thought that the more varied the sound, the better (a56).

Although many people viewed diversity as positive, others felt that too many kinds of sounds can confuse listeners. Some people expressed that too many sounds mixed at a time can be noisy. They mentioned that no more than three kinds of sounds are acceptable (a64, a96). Others accepted more types of sounds as long as they ‘*mix well together*’ ‘*in a natural way*’ (a28, a57). Thus, they stressed ‘integrality’ (a60, a43). For the sake of this wholeness, particularly ‘harsh’ sounds, such as sudden sounds (alarm, brake) and high-volume sounds (loud music, traffic) (a19, a86), were considered to require improvement.

#### 3.2.2. Particularity and Stereotypes

People desired that the square soundscape be unique, but at the same time, they required it to conform to the stereotypical attributes of a square. One of them did not like store music because it was too popular to be featureless (a20). The sound of water was praised as it represented Sheffield’s character. The ubiquitous water sounds were believed make the city more memorable (a16, a18, a19).

On the contrary, participants had limited imagination regarding what constitutes a general square soundscape. They displayed a similar, uniform understanding of the square soundscape, for instance, ‘*Sound types in this public space are all basic sounds, very common; fountain, children, talking are ordinary sounds in the public space*’ (a75, a42). Interviewees mentioned that they wanted the other environmental factors (light, temperature, sanitations) to match the sound environment. In return, they did not ask for a perfect sound environment. Instead, they considered commonness to be even better (a50). As a result, some unusual sounds were considered unacceptable, such as loud music. Loud music was recognised as ‘*a sound only found in pubs*’ (a86), and therefore inappropriate in the square. People might wish for each place to perform its functions and to have its standards. To sum up, stereotypes and particularity are not contradictory: what people want is the particularity within their square stereotype.

### 3.3. Psychological Reactions to Sounds

Sounds can bring about psychological reactions, and participants tended to describe the soundscape by describing the feelings that sounds triggered. A soundscape was found to bring about two kinds of subjective reactions: (1) instant and transient and (2) relatively stable and prolonged. In response to sound-induced subjective reactions, especially negative ones, participants adopted strategies of tolerance, avoidance, and complaint.

#### 3.3.1. Instant Reactions

Instant psychological reactions were found to be triggered by particular sounds in the square: (1) happy/depressed: *speaking, birds’ singing, children screaming* (a3, a48, a77), *skyscraper effect sound, wind* (a40); (2) awkward: *others’ conversation, music on others’ phones* (a91, a98); (3) relaxed, calming, peaceful: *waterfall, fountain, sounds representing nature* (a80, a13, a55, a22); (4) unsafe, worrying: *car brake, bus* (a24, a99); (5) energetic, exciting, lively, vivid: *dancing music, children playing and screaming* (a38, a30); and (6) sociable: *festival music* (a14). In addition to the particular sound source, the visual aspect was tightly combined with sounds in the aspect of triggering a particular feeling. For example, the image of children playing accompanied by the sound of children screaming triggered a happy feeling. Meanwhile, the interviewee would not feel happy if they only heard screams (a77).

#### 3.3.2. Prolonged Reactions

Prolonged psychological reactions are relatively stable compared to the instant reactions. For example, unlike the instant feeling of ‘*quiet*’, the feeling of ‘*tranquillity*’ is long and stable (a77). In addition, as public squares are mostly located in city centres, people mentioned that they felt depressed (a34)/depressed (a90) when facing all the high-rise buildings (a34). This feeling was not triggered by particular sounds, but was related to their living conditions. By contrast, the sounds of nature eased anxiety. People mentioned that they came to the squares to experience nature and to get rid of their working space (a29, a47, a80, a83).

#### 3.3.3. Responses and Strategies

Psychological reactions were found to influence both mental and physical aspects. Calm sounds can made people think, for example, rainy sounds (a49). Happiness, on the contrary, was associated with physiologically perceived warmth (a66). Similarly, a calm and tranquil feeling brought about by the water sound was believed to reduce the temperature in hot days (a22).

Positive feelings were found to provide restoration and benefit mental health. Fountain sounds were found to bring positive feelings and relieve people from stress (a69). Public spaces with large areas of greenery can reduce urban noise and bring with them calmness (a25). While, the consequences of negative feelings were more far-reaching and severe, with potential long-term negative effects on psychological and physical aspects. Anxiety aroused by traffic noise made people physically uncomfortable (a52). The sounds of loud laughter and shouting from groups of teenagers cast a long psychological shadow over one of the participants (a32).

Three strategies for coping with negative emotions were summarised from the interviews: tolerance, avoidance, and complaint. Tolerance was adopted when people felt that they could not change the situation, and they finally accepted it. Such strategies were often adopted in response to traffic noise, which people considered unavoidable in cities (a94, a76, a93). When people felt that they could not cope with unwanted sounds, they would choose avoidance. Some of them considered it a way to control the situation as they thought they had the option to leave. As long as they could leave the place at any time, they felt everything was under control, and they would not feel stressed about the unwanted sounds anymore (a39, a92). Others chose to complain about negative sound experiences (such as posting on the website), and they gave suggestions in an attempt to improve the future sound environment (a55); (a31, a63). In short, these three strategies cover people’s psychological adaptation. When people were met with noise problems, they tended to solve problems at the psychological level.

### 3.4. Soundscape Preferences

People were found to express their preferences for the total acoustic environment in two ways: through adjectives or descriptive phrases and through ‘image’ description—expressing soundscape preferences by narrating an event in the square, in which sounds appeared as a part of the ‘image’.

#### 3.4.1. By Descriptive Words

People used phrases or adjectives that directly point to the preferred/annoyed sound source or preferred/annoyed feelings brought about by sounds. ‘Preferred’ or ‘annoying’ is an essential measurement by which people understand and evaluate sounds. It was also found that soundscape preferences contained judgment about their preferences for the three aspects mentioned above: sound sources, features, and psychological reactions. As shown in Table 4, descriptive words were categorised according to the three aspects from positive and negative perspectives. Generally, positive sound sources, features, and psychological reactions were preferred, especially for the human and nature sounds (children playing (a77), talking (a48), birds singing (a51, a53), fountain (a11), water (a83), and music (a69)). People described their preferred sound sources as beautiful (a72), natural (a29), and quiet (a104). Most of the annoying sounds were under the categories of city and human sounds, described as noisy/loud and artificial, including traffic/cars (a99, a24, a52), vendor sounds (a26), shop music (a20), square dancing music (a102), loud talking (a63), and children screaming (a42). Although most people disliked hearing loud sounds, some could accept a reasonable level of loudness in the square, considering the context (a44). Some people even expressed a preference for loudness, considering it an indicator of eventfulness (a76). In the context of conveying information, people tended to prefer meaningful sounds (a18).

Preferences about soundscape features were ‘various’ (a56, a57) and ‘harmonious/united’ (a28) in the aspect of diversity and integrality and ‘distinctive’ (a20), ‘typical’ (54), and ‘appropriate’ (a86) for particularity and stereotypicality. The preferred psychological reactions were mostly those associated with positive emotional feelings, such as happiness and relaxation.

#### 3.4.2. By Describing an ‘Image’

When people felt that it was challenging to describe the preferred soundscape, they described an ‘image’, including information of ‘who’, ‘where’, and ‘doing what’. The word ‘image’ was originally mentioned by one of the interviewees when she described how she had a great soundscape experience with her children in Peace Garden (a77). ‘Image’ referred to the phenomenon that respondents tended to describe their expectations and desires for soundscapes based on their life experiences and knowledge of the world. Various pieces of socio-demographic information were included in these ‘images’, such as occupation, age, gender, and social relationships. Social relationships were dominant, as activities were centred around them. People’s activities were found to correspond to their social relationships. Categorised by social relationships, three types of ‘images’ emerged: those pertaining to friends, family, and couples. People mentioned an ideal ‘friend image’ with an eventful soundscape in relation to vacation, festivals, and performances (a14); (a97). The ‘family image’ involved a soundscape that was ‘*relaxing, peaceful*’ (a4); and ‘*nature sounds*’ ‘*accompanied*’ (a85). The ‘couples image’ was generally concerned with quietness and a private sound environment (a91, a13, a98). In these images, soundscape preferences also included judgments of the above three categories, such as relaxing (psychological reactions), nature sounds (sound classification), and hearing sounds not belonging to the square (stereotype).

Socio-demographic information is usually considered to influence people’s soundscape preferences [32]. In the ‘image’, socio-demographic information provided the background as to how those activities occurred. To some extent, it explains why demographic information influences soundscape preferences. According to the content of the ‘image’ illustrated above, it was found that people’s social attributes determined what kind of activities they participated in. Especially for social relationships, there was a strong connection between relationship type and activity type. Apart from personal preferences, people preferred sounds that supported and stimulated their activities. In short, social attributes influence people’s soundscape preferences through the sound requirements for their activities.

### 3.5. A Perceptual Structure of Soundscape: The Process of Perceiving Sounds

**Figure 1:**
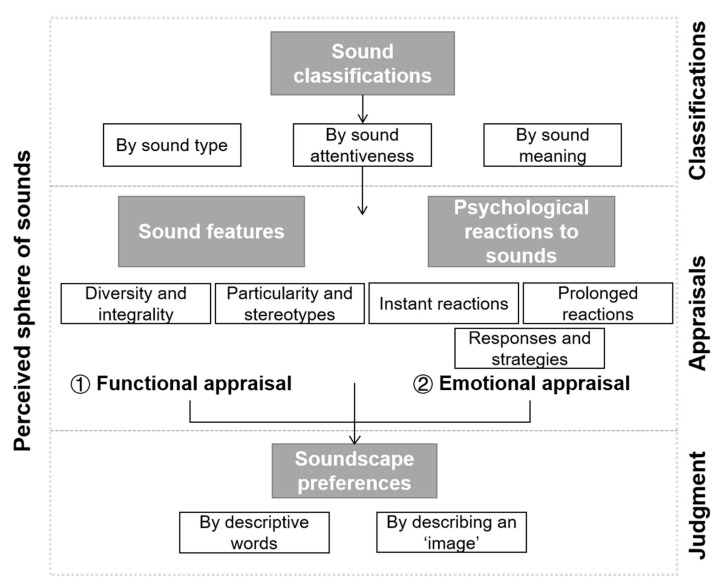
Perceptual structure of soundscapes.

The perceptual structure of soundscapes includes perception aspects and the perceiving process. According to Figure 1, four aspects make up the perceived sphere of sounds: sound classifications, features, psychological reactions, and preferences, forming three levels of perceiving process: classification, appraisal, and judgment. When sounds reach people’s ears, they express what they hear through classification. This step is the starting point from physical sounds to the sphere of perception, and it provides the basis for soundscape features and psychological reactions. Based on the classifications, people appraise sounds through two methods: one is a rational and functional appraisal, which evaluates the features of sounds, and the relationships between the single sound and the overall sound environment; the other is from the emotional aspect, emphasising the feelings and emotions triggered by sounds. In this study, the two appraisal methods emerged at the same time. Some participants only appraised sounds from one perspective, while others appraised them from both perspectives. At the final level, soundscape preferences reach the value judgment level, with the preferred-annoyed criteria to judge the previous three aspects. Thus, a progressive process of sound perceptions was derived: classification—appraisal (sound features and psychological reactions)—judgment (soundscape preferences).

The perceptual structure stresses two points about the soundscape: first is that there is a hierarchy in people’s perceptions of sounds, whereby the four aspects form three progressively more profound levels of sound perceptions; second is that soundscape preferences entail value judgment about the sound classification, features, and psychological reactions through descriptive words and narrative ‘image’.

## 4. Discussion

The perceptual structure generated in this study has associations with previous research in the environmental psychology field. The process of perceiving sounds corresponds to Rapoport’s [33] process of how people perceive the physical environment, which consists of the cognitive, affective, and conative levels, that is, knowing something, feeling something, and then doing something about it. The four aspects of the perceptual structure correspond to three levels: sound classifications and features represent how users receive and understand sounds at the cognitive level; psychological reactions are at the affective level, including feelings and emotions stimulated by the sound environment; preferences reach the level of judgment and choosing, which represent the conative level.

Three levels of perceiving the physical environment are also seen in Morris & Boulding’s [34] ‘image’ theory. They referred to the ‘image’ as one’s subjective knowledge of the world, one’s sense of being located in space and time, and in a web of human relations and emotions. People’s behaviours are dependent on their images of the world. This corresponds to the phenomenon that interviewees tended to describe their soundscape preferences through describing an ‘image’ consisting of social relationships, events, and space/time. People’s sound perceptions are embedded in their subjective knowledge of the world, which can be expressed through the ‘image’ [34,35]. Further, Morris and Boulding pointed out that the ‘image’ comprises what one knows and thinks about an object (cognitive level), how one feels about it (affective level), and how one acts using this information (conative level). In other words, image theory confirms that soundscape preferences are complex enough to contain all three levels of perception. It echoes the perceptual structure of this study, where soundscape preferences contain judgment of the previous three aspects.

Social relationships are the dominant aspect of the ‘image’ of sound because they influence people’s soundscape preferences through the activities they engage in. People tended to require the soundscape to support their activities. This corresponds to Gibson’s [36] affordance theory, which referred to the quality of an object or an environment that supports the performance of an activity. In this study, activities and social relationships were closely combined. Sound requirements seemed to afford not only participants’ activities but also their relationships. Further studies may be needed to enrich the meanings of affordance in this light.

## 5. Conclusions

This study aimed to explore the aspects of the perception process in order to build a perceptual structure of soundscapes for public space users. Based on the grounded theory approach, four aspects were summarised: sound classification, features, psychological reactions, and preferences. (1) Sound classification: the way people categorise sounds reflects the fundamental understanding of sounds. Ordinary listeners tended to categorise sounds by content and sound levels. (2) Sound features: people can think dialectically about the relationship between individual sound and the overall soundscape. (3) Psychological reactions to sounds: sounds trigger instant or prolonged psychological reactions, which can result in physical and psychological outcomes in listeners. To deal with the negative outcomes, people adopt the strategies of tolerance, avoidance, and complaint. (4) Soundscape preferences: people were found to express preferred sounds by descriptive words and narrative ‘images’. ‘Image’ preference indicates the approach towards perceiving the physical world. The dominant status of social relationships found in the ‘image’ reflects the social attributes of people in the square’s activities. Social relationships influence sound preference through people’s sound requirements for different activities.

Based on the previous studies, especially the ISO’s perceptual construct of soundscape, this study focused on the aspects and process of the urban public space users perceiving, understanding, and experiencing sounds from the insight of sociology. The perceptual construct of ISO is based on the psychological theory, which explains how humans respond to the external stimulus with feelings and responses/behaviours. Sound is understood as one of the external stimuli, and soundscape exists through human perception of the acoustic environment. The perceptual structure of soundscape summarised in this study expanded the ISO’s structure to explore how a socially constructed listener transforms auditory signals into sound awareness. Four soundscape aspects categorised by three levels of perceiving progress make up the perceived sphere of sounds: classification—sound appraisals (sound features and psychological reactions)—judgment (soundscape preferences). Sound classification represents a basic understanding of sounds. Appraisals involve functional and emotional evaluations of sounds, representing rational and emotional thinking. In the end, soundscape preferences are generated based on the knowledge of the previous three aspects. The two descriptive methods of soundscape preferences reflect listeners’ desires and expectations for soundscape based on their social relationships, social roles, social status, etc.

The four perceptual aspects summarised in the GT approach provide a comprehensive view of sound perceptions. The three-level perception process offers the possibility of analysing soundscapes from various perspectives. During the judgment, two methods of describing soundscape preferences were found in this study, descriptive words and narrative ‘images’. These two methods expand the scope of soundscape preferences and evaluations. For example, in soundscape studies that aim to simulate the urban public space environment in laboratories, it may be possible to better recreate the scene of public spaces by carrying out some activities. Social relationships emphasised the preference for ‘image’, and the influence of companionship should be explored in future studies. However, owing to the limited human resources, this research neither included various kinds of urban public spaces, nor covered a variety of seasons; thus, the perceptual structure of soundscapes derived from this study may not be generalisable to all kinds of urban public spaces under all conditions. The factor of season can influence the activities of humans and animals in urban public spaces, thus affecting the acoustic environment. Further studies in other kinds of urban public spaces across the different seasons are required to enrich this structure. Additionally, this study involved different urban public spaces across the countries, which brought variances in site context. The site and cultural differences were not adequately compared and analysed as this study was concerned with the dimensions and overall process of how the public space users perceive sounds. The selected sites with cultural differences were intended to increase the sample’s diversity so as to dig deep into the perceptual sound sphere, rather than to make case comparisons. Future research must attempt to fill this gap.

## Figures and Tables

**Table 1:** Demographic information of the recruited respondents.

Site	Respondent Number	Gender		Age		
		Male	Female	Young	Adult	Older
Guanqian Squre, Suzhou	5	3	2	3	3	1
Central Park Square, Suzhou	5	5	0	0	3	2
Peace Garden, Sheffield	9	3	6	3	4	2
Barker’s Pool, Sheffield	4	2	2	2	2	0
Total	23	13	10	6	12	5

**Table 2:** The structure of interview.

Category	Question Details	Question Aims
Background information	(a) Age and occupation through questions, gender through observation	Demographic background
	(b) Whom have you come with today? With whom do you generally go to a public space?	Companion types
	(c) Why have you come to this place? What are you doing here? Do you often come here for this activity?	Activity types
Descriptions of sounds	(a) How do you think about this place? What are the physical environmental factors you care most about an urban public space? How would you describe the preferred environment in a public space? Why? (b) Do you pay attention to sounds? How do you think of sounds compared to other factors?	Overall impressions about sounds as part of environmental factors in general
	(c) What sounds do you hear in this place? How do you think of these sounds? How would you describe them?	Overall impressions about sounds at the site
Subjective understandings	(a) What do you feel about those sounds? What sounds do you like or dislike? Do you feel the soundscape in the square hinders/stimulates your activity?	Subjective evaluations of current soundscape
	(b) Describe your preferred soundscape in urban public spaces? Or describe a place you have been to that had a great soundscape? What have you heard in this public space that you have liked? What kind of improvements would you like to see in the square soundscape?	Experiences about public space soundscapes

**Table 3:** Semantic coding analysis based on the grounded theory: open, axial, and selective coding.

Sorting Memos	Labelling	Conceptualising Data	Categorising Data	Categories	Sub-Categories
(‘Why have you come to this place? What are you doing here? Do you often come here for this activity?’) ‘I use it as a base when I’ve been *working*, and then *relax* out in the square…’ ‘Maybe because it is like the *main spot* in the city, like, everybody comes here *together*.’ ‘It’s very *central*, we just get off the train and a little walk … usually we come here to take a *rest* on the way to the bank or shopping...’ ‘*Playing with water*, which *children* like’. ‘I would love to … *fountain* … and something to do with *nature* … like water, green, somewhere like here ….Very *relaxing*, and the *kids* are having fun around the fountains’. (‘What are the physical environmental factors you care most about in a public square? How would you describe the preferred environment in a public space? Why?’) …	a1. Location affects whether people come here a2. Many people come here because of the square’s central location a3. People like to see other people a4. People usually take children to the fountain a5. The fountain makes people feel relaxed a6. Open spaces, greenery, and fountains are important for public spaces a7. People prefer things involving nature a8. Public spaces exist for relaxation …	aa1. The weather is the reason people come to the public space; sound is the second priority. (a9, a13, a16, a75, a36) aa2. Centrality is the main feature of public spaces. Numerous people come to the public space. (a1, a2) aa3. People love to see and hear other people in public places. (a3, a91, a98) aa4. Children like playing in the fountains. Families always take their children to the fountains. (a4, a5, a6) aa5. Open space and nature are preferred. (a6, a7, a39) …	A1. A comfortable environment is a more important reason for coming to public spaces than sounds. (aa1, aa12) A2. People love to see other people and hear others’ conversations. This makes them feel happy and enjoy the space. Talking sounds are a main feature of public spaces. (aa3, aa48, aa54) A3. Hearing others’ conversations is awkward. People don’t want to hear others playing music on their phones. (aa91, aa98) A4. Public spaces are centrally located and have various modes of transportation. Some people do not go there on purpose. They just go past it or rest there. (aa2, aa21, aa26, aa61) A5. Annoying surrounding sounds will damage the quality of public spaces. ...	AA1. Sound classifications AA2. Sound features AA3. Psychological reactions to sounds AA4. Soundscape preferences	AA1. Sound classification: - By sound type - By attentiveness - By sound meaning AA2. Sound features: - Diversity and integrality - Particularity and stereotypicality AA3. Psychological reactions to sound: - Instant reactions - Prolonged reactions - Responses and strategies AA4. Soundscape preferences: - By descriptive words - By describing images
Initial Data	176 items	105 items	55 items		

**Table 4:** Descriptive words for preferred and annoyed sounds in urban public spaces.

	Preferred	Annoying
Sound classifications	Quiet	Noisy/loud
	Meaningful	Meaningless
	Memorable	Forgettable
	Natural	Artificial
	Beautiful	Tuneless
Sound features	Varied	Monotonous
	Harmonious/united	Conflict
	Distinctive	Ordinary
	Typical	Unusual
	Appropriate	Inappropriate
Psychological reactions to sounds	Happy	Depressed
	Relaxing/relieving	Stressful
	Tranquil/calm/peaceful	Exciting
	Eventful/energetic/lively/vivid	Dull
	Warm	Cold
	Thoughtful	Shallow
	Safe	Unsafe
	Comfortable	Awkward
	Unconcerned	Worried
	Calm	Irritated
	Fearless	Fear
	Polite	Offensive

## Data Availability

The data presented in this study are available on request from the corresponding author. The data are not publicly available due to the privacy of participants.
